# Massively parallel sequencing of 231 autosomal SNPs with a custom panel: a SNP typing assay developed for human identification with Ion Torrent PGM

**DOI:** 10.1080/20961790.2017.1281011

**Published:** 2017-02-14

**Authors:** Suhua Zhang, Yingnan Bian, Anqi Chen, Hancheng Zheng, Yuzhen Gao, Yiping Hou, Chengtao Li

**Affiliations:** a Shanghai Key Laboratory of Forensic Medicine, Shanghai Forensic Service Platform, Institute of Forensic Sciences, Ministry of Justice, PRC, Shanghai, China; b State Key Laboratory of Genetic Engineering, Institute of Genetics, School of Life Sciences, Fudan University, Shanghai, China; c Department of Forensic Medicine, Medical College of Soochow University, Suzhou, China; d Department of Forensic Genetics, West China School of Preclinical and Forensic Medicine, Sichuan University, Chengdu, China

**Keywords:** Forensic science, forensic genetics, single nucleotide polymorphism (SNP), massively parallel sequencing (MPS), Ion Torrent personal genome machine (Ion Torrent PGM)

## Abstract

The custom-designed single nucleotide polymorphism (SNP) panel amplified 231 autosomal SNPs in one PCR reaction and subsequently sequenced with massively parallel sequencing (MPS) technology and Ion Torrent personal genome machine (PGM). SNPs were chosen from SNPforID, IISNP, HapMap, dbSNP, and related published literatures. Full concordance was obtained between available MPS calling and Sanger sequencing with 9947A and 9948 controls. Ten SNPs (rs4606077, rs334355, rs430046, rs2920816, rs4530059, rs1478829, rs1498553, rs7141285, rs12714757 and rs2189011) with low coverage or heterozygote imbalance should be optimized or excluded from the panel. Sequence data had sufficiently high coverage and gave reliable SNP calling for the remaining 221 loci with the custom MPS–SNP panel. A default DNA input amount of 10 ng per reaction was recommended by Ampliseq technology but sensitivity testing revealed positive results from as little as 1 ng input DNA. Mixture testing with this panel is possible through analysis of the *F*_MAR_ (frequency of major allele reads) values at most loci with enough high coverage depth and low level of sequencing noise. These results indicate the potential advantage of the custom MPS–SNP assays and Ion Torrent PGM platform for forensic study.

## Introduction

Within forensic community, standardized methods of PCR amplification and capillary electrophoresis (CE) of short tandem repeats (STRs) are usually applied in forensic laboratories [1,2]. However, forensic casework DNA samples are often of insufficient quantity or quality to generate full profiles with this conventional typing method [3–5]. Amplification of STRs is inherently limited in samples containing degraded DNA, as the cumulative size of repeat regions, primer binding regions, and flanking sequence is sometimes larger than 200 bp [2–4]. Single nucleotide polymorphism (SNP) or insertion/deletion (Indel) markers may yield more information from challenging samples due to their smaller amplicon length [5]. Additionally, CE assay designing limits the number of detected markers because they must be separated by size within each dye colour; however, the number of dye colours was limited by the instrument optics [1–4]. Massively parallel sequencing (MPS) have the potential to genotype hundreds of STRs or SNPs in large numbers of samples analyzed in parallel without restriction of dye-labelling [6–8]. Till now, two commercial kits were available for sequencing of SNP loci selected for human identification with MPS technology: the HID-Ion Ampliseq™ Identity Panel (currently sold under the name of “Precision ID Identity Panel” by Thermo Fisher Scientific) [9] and the ForenSeq DNA Signature Prep Kit (Illumina) [10]. Both kits type most of the SNPs in the SNPforID [11] and IISNP [12]. These commercial panels and accordingly MPS platforms provide a sensitive and accurate forensic SNP genotyping assay. Evaluation reports of the two panels [9,10] reveal that there is still a need for supplementary DNA marker typing in order to increase the power to solve cases for both individual identification and complex kinship issues (e.g. half-sibling testing without parental information, relationship between uncle and nephew without reference samples, etc.).

In the present study, a custom SNP panel containing 231 forensic polymorphic SNPs covering 22 human autosomes was sequenced using Thermo Fisher Ion Torrent PGM system. Libraries were constructed using Ampliseq technology. The MPS–SNP panel was evaluated by concordance study, analysis of population samples, sensitivity testing, as well as mixture testing.

## Materials and methods

### Panel designing

In this study, public SNP databases of HapMap (http://hapmap.ncbi.nlm.nih.gov/), dbSNP (http://www.ncbi.nlm.nih.gov/snp/), and related published literatures [11–20] were involved.

The following steps of SNP screening were applied:

1. Universal SNPs included in SNPforID [11] and IISNP [12] with minor allele frequency (MAF) above 0.1 in Chinese Han population [9] were included.
2. SNPs reported with MAF > 0.3 in Han population of China or Asian populations by extensive studies [13–20] were included. SNPs should follow Hardy–Weinberg equilibrium (HWE).
3. HapMap (#28, Phases 2 and 3) was adopted for genome-wide SNP screening. SNPs with MAF above 0.3 in Beijing Han population of China (CHB) and show little frequency variation (Fst < 0.06) among main populations (African American, European American, and East Asian) were included. SNPs should be located in the intron area and follow HWE.
4. All selected markers should keep a minimum distance of 5 Mb from each other and most commonly used forensic STR loci (listed in STRBase website). SNPs should locate away from copy number variants (CNV) regions, which are defined in the Database of Genomic Variants (http://projects.tcag.ca/variation/); have appropriate GC content (between 30% and 60%) in the flanking sequences; and have no long homopolymers (>5 nt) or Indels in the flanking sequences of targeted SNPs (±20 bp).

A formatted BED file including the final candidates was submitted to Life Technologies’ AmpliSeq primer designing tool (http://www.ampliseq.com) for multi-primer designing. And a single pool with amplicon length of 125–175 bp which is suitable for degraded DNA testing was provided and chosen for the following study.

### Samples, library construction and template preparation

Two-hundred milliliter of peripheral blood samples from 50 unrelated healthy Chinese Han individuals were collected, with the approval of Ethics Committee of Institute of Forensic Sciences, Ministry of Justice, PRC. Informed consent was obtained from each participant. DNA was extracted using QIAamp® DNA Blood Mini Kit (Qiagen, Hilden, Germany) [21]. DNA was quantified by Quantifiler Human DNA Quantification Kit (Thermo Fisher Scientific, USA) with 7500 Real-time PCR System (Thermo Fisher Scientific, USA) [21] and diluted to 10 ng/µL. One microliter of the prepared initial DNA was recommended by Ampliseq Designer for library construction. Detailed information of samples is listed as Supplementary Table S1.

Control samples of 9947A and 9948 (Promega, Wisconsin, USA) were adopted as reference samples for concordance and accuracy testing, and Sanger sequencing was adopted as validation method. The two libraries were pooled together and sequenced on Ion 314 chip in duplicate. Serial dilutions of control DNA 9948 were performed to generate DNA concentrations of 10, 5, 2, 1, 0.5 and 0.2 ng/µL for sensitivity testing. And 1 µL of each concentration was added in the library construction PCR system, thus the DNA input for sensitivity testing ranged from 10 to 0.2 ng. Libraries of the six dilutions were sequenced in parallel with Ion 316 chip in duplicate. Two reference samples (9947A and 9948) with ratios of 100:1, 10:1, 5:1, 1:1, 1:5, 1:10 and 1:100 were prepared for mixture study. For the 1:1 ratio, 5 ng of each DNA was mixed together. For the ratio of 100:1 and 10:1, 50 and 500 pg of 9948/9947A were added to 5 ng of 9947A/9948, while for the 5:1 ratio, 2.5 ng of 9947A/9948 was mixed with 500 pg of 9948/9947A. Therefore, the DNA input ranged from 3 to 10 ng for library-construction system and libraries of the seven mixtures were sequenced with Ion 316 chip in duplicate.

Ampliseq technology was adopted for library construction. Chemistry of Ion AmpliSeq Library Kit 2.0 (Thermo Fisher Scientific, USA) and the primer pool including targeted-SNP primer pairs (307 µmol/L, each) were used for library preparation. The total library-PCR system was 20 µL, with 4 µL of 5× Ion AmpliSeq HiFi Master Mix, 10 µL of 2× custom primer pool, 1 µL of above prepared DNA and 5 µL of nuclease-free water. The library-PCR procedures were as follows: 2 min at 99 °C, 17 cycles of 15 s at 99 °C and 4 min at 60 °C followed by a 10 °C hold. According to our pre-testing results, 22 PCR cycles were adopted when library DNA amount was lower than 1 ng. The amplicons were treated with 2 µL FuPa reagent (Thermo Fisher Scientific, USA) to partially digest primers with incubation for 10 min at 50 °C, 10 min at 55 °C, 20 min at 60 °C and hold up to 1 h at 10 °C. Library-barcoding was labelled with the Ion Xpress™ Barcode Adapters (Thermo Fisher Scientific, USA) and library purification was performed with Agencourt AMPure Reagents (Beckman Coulter, Brea, CA).

Ion Library TaqMan Quantitation Kit (Thermo Fisher Scientific, USA) on 7500 Real-time PCR System (Thermo Fisher Scientific, USA) was adopted for accurate library quantification. All library samples of a run were pooled in equimolar prior to sequencing. Pooled libraries were subjected to emPCR on Ion OneTouch™ 2 instrument (Thermo Fisher Scientific, USA) with the Ion PGM™ Hi-Q™ OT2 Kit (Thermo Fisher Scientific, USA), and cycling setting was selected as “PGM: Ion PGM™ Template OT2 200 Kit for Hi-Q™”. The optimal amount of library corresponds to the library dilution point that gives percent of ion sphere particles (ISPs) was between 10% and 30% [9]. Template-positive ISPs were enriched on the Ion OneTouch™ ES instrument (Thermo Fisher Scientific, USA) according to the manufacturer's recommendations.

### Sequencing and data analysis

Parallel sequencing was performed on Ion Torrent PGM with Ion PGM™ Hi-Q™ Sequencing Kit. Sample preparation and sequencing chip information are listed in Supplementary Table S1. Raw data were collected as DAT files and signal processing, base-calling and barcode de-convolution were processed with Ion Torrent Suite Server v4.4.0 (Thermo Fisher Scientific, USA). Homo genome of GRCh37/Hg19 was adopted as reference sequence. Coverage Analysis (v4.0-r77897), HID_SNP_Genotyper.42 (v4.2), and Variant Caller (v4.0-r76860) plug-ins with default settings were applied for data analysis. BAM files were checked with IGV_2.3.59 software. And parameter of *F*_MAR_ (frequency of major allele reads) was adopted to evaluate the performance of SNP calling, both for heterozygotes and homozygotes. *F*_MAR_ was calculated as the biggest reads among the four bases (A, C, G and T) dividing the total detected reads at the base position. According to our previously published study [9], *F*_MAR_ for accurate heterozygotes calling should range from 50% to 60%, while for homozygotes, it should be above 90%.

Arlequin 3.5 [22], PowerMarker v3.25 [23] and SNPAnalyzer were adopted for HWE testing, linkage disequilibrium (LD) analysis and forensic parameters estimation of the included SNP targets.

## Results

### SNPs selection and panel designing

In this study, 129 polymorphic universal SNPs from database of SNPforID [11] and IISNP [12] with MAF > 0.1 in Chinese Han [9] were included first. With published references of [13–20], 25 new SNPs with MAF > 0.3 in CHB or Asian population were found. Three SNPs (rs1029047, rs3808378 and rs1108414) of them were located within long homopolymers with sequence-checking and should be excluded from the panel. And with HapMap database (#28, Phases 2 and 3), 29,530 SNPs located in intron with MAF > 0.3 in CHB were obtained. Subsequently, 936 SNPs were retained after selection based on genotype frequency data with HWE (*P*>0.05) and Fst < 0.06 which performed with population difference testing among African American, European American, and East Asian populations. After applying the location and sequence criteria detailed in Part 2.1, 85 auto-SNPs were left as candidate targets. Until this point, a total of 236 auto-SNPs were chosen and accordingly formatted BED file of them were submitted to Thermo Fisher AmpliSeq primer design online tool. An approach including 231 SNPs with amplicon length of 125–175 bp was provided. Five SNPs were deleted from the final panel, as either the primer designing was difficult or the primer was not compatible with others. Detailed information including chromosomal position, unmodified primer sequences, start and stop position of these final targets is listed in Supplementary Table S2. Primer sequences were modified by the manufacturer (Thermo Fisher Scientific, USA) using a proprietary method, prior to being synthesized for library construction with the AmpliSeq Library Kit.

### Concordance study

Control samples of 9947A and 9948 were used for concordance study. MPS results were compared with Sanger sequencing. Unmodified primers in Supplementary Table S2 were used for Sanger sequencing. HID_SNP_Genotyper.42 (v4.2) plug-in and Chromas software were used for genotyping of MPS data and Sanger sequencing data. All results of 9947A and 9948 are listed as Supplementary Table S3-1 and Supplementary Table S3-2, respectively.

In Supplementary Table S3-1, coverage depth of rs1498553 (r143), rs1478829 (r087), rs7141285 (r174), rs2189011 (r102) and rs12714757 (r043) were below 100× and coverage depth of rs1498553 (r143) is only 2× which is not available for genotyping calling. *F*_MAR_ values of heterozygotes at rs430046 (r192) and rs4606077 (r116) were between 60% and 90%. In Table S3-2, the same five SNPs (rs1498553 (r143), rs1478829 (r087), rs7141285 (r174), rs2189011 (r102) and rs12714757 (r043)) were detected with coverage depth below 100× and rs1498553 (r143), rs1478829 (r087), rs7141285 (r174) were even detected with coverage depth below 20× and labelled with “NN”. *F*_MAR_ values of heterozygotes at rs4530059 (r178), rs334355 (r123) and rs2920816 (r157) were between 60% and 90%. However, no inconsistent result was observed between available MPS calling and Sanger sequencing.

The noise ratio was calculated as the number of reads with nucleotide calls that differed from the SNP genotypes (base call error) dividing the total reads. Supplementary Figure S1 lists the noise ratio data of 9947A and 9948 at the 231 SNPs. The noise ratio range was from 0% to 4.1041% for 9947A, while it was from 0% to 6.4796% for 9948.

### Sequencing performance

Fifty blood samples of unrelated individuals were typed twice with the custom panel. Libraries of 10 ng initial DNA were prepared and sequenced for intensive study. Table 1 lists 10 problematic SNPs either with lower coverage or with heterozygote imbalance (*F*_MAR_ values 60%–90%). SNPs of rs1478829 (r087) and rs1498553 (r143) were detected with heterozygotes imbalance and lower coverage reads (<20), indicating the designed primers are insufficient and improper. For the three SNPs of rs7141285 (r174), rs12714757 (r043) and rs2189011 (r102), lower coverage reads with ideal allelic performance were obtained, suggesting improving the concentration of primers in the library pool may optimize sequencing performance.

**Table 1.** Problematic SNPs found with the custom MPS--SNP panel by sequencing 50 unrelated individuals.

Panel No.	SNP	Performance
r116	rs4606077	Heterozygote imbalance (mean *F*_MAR_% = 63)
r123	rs334355	Heterozygote imbalance (mean *F*_MAR_% = 79)
r192	rs430046	Heterozygote imbalance (mean *F*_MAR_% = 78)
r157	rs2920816	Heterozygote imbalance (mean *F*_MAR_% = 71)
r178	rs4530059	Heterozygote imbalance (mean *F*_MAR_% = 64)
r087	rs1478829	Heterozygote imbalance (mean *F*_MAR_% = 68) and mean coverage reads < 20
r143	rs1498553	Heterozygote imbalance (mean *F*_MAR_% = 75) and mean coverage reads < 20
r174	rs7141285	Mean coverage reads < 100
r043	rs12714757	Mean coverage reads < 100
r102	rs2189011	Mean coverage reads < 100

**Figure 1.**
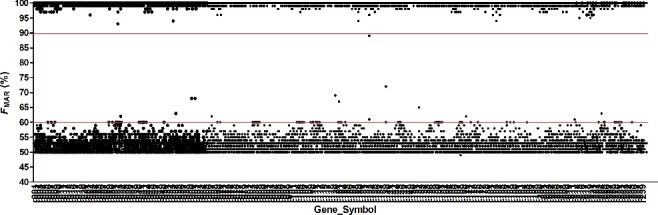
Allelic performance of the 221 targeted SNPs were evaluated with the parameter of *F*_MAR_ (10 problematic SNPs in Table 1 were excluded). *F*_MAR_ data was calculated as the biggest reads among the four bases (A, C, G and T), thus dividing the total detected reads at the base position. The *F*_MAR_ for accurate heterozygotes calling should be 50%–60%, while for homozygotes, the calling should be 90%–100%. The order of the SNP Gene_Symbol is consistent with that in Table S2.

The 10 problematic SNPs in Table 1 were excluded from the following analysis. Allelic performance of remaining 221 targets with the two separate sequencing is shown in Figure 1. Except several genotypes, all the *F*_MAR_ values of heterozygotes distributed between 50% and 60%, and of homozygotes were above 90%. Several genotypes were observed with *F*_MAR_ values between 60% and 90%, probably caused by occasional sequencing effect. The mean coverage depth and the median coverage depth among the 221 SNPs were 2 532× and 2 755×, respectively, while the lowest average coverage was 1 163× at rs1454361 with a standard deviation (SD) of 466.8, as well as the highest coverage was 4 150× at rs2730648 with SD of 1 940. Mean noise ratio ranges from 0% to 5.8% (rs10495407).

### Sensitivity testing

Libraries of series dilutions (S1: 10 ng, S2: 5 ng, S3: 2 ng, S4: 1 ng, S5: 0.5 ng and S6: 0.2 ng) of a control male DNA 9948 were prepared. When initial DNA was above 1 ng, recommended PCR cycle of 17 was adopted; and when DNA was equal to or lower than 1 ng, five more cycles were added for library-construction PCR. The six libraries were sequenced on Ion 316 chip in duplicate.

**Figure 2.**
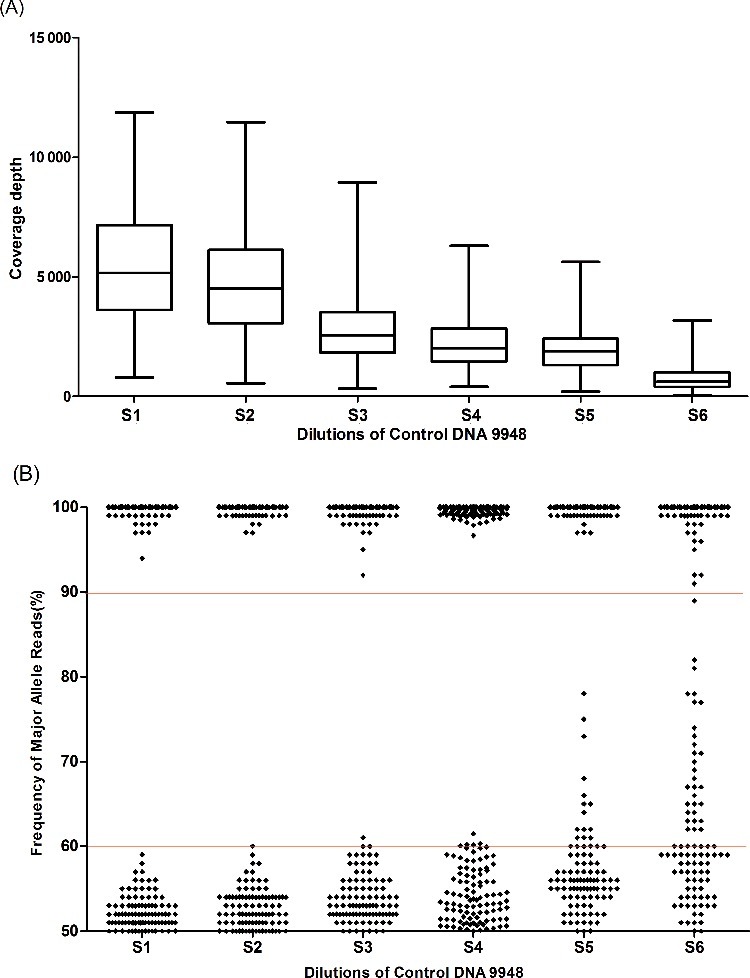
Sensitivity testing of series dilutions (S1: 10 ng, S2: 5 ng, S3: 2 ng, S4: 1 ng, S5: 0.5 ng and S6: 0.2 ng) of control DNA 9948. (A) Coverage reads of series dilutions; (B) allelic performance of 221 SNP of series dilutions, and 10 problematic SNPs in Table 1 were excluded.

The calling rate of S1 (10 ng) to S5 (0.5 ng) was always 100%, and of S6 (0.2 ng) was 98.64% with the analytical threshold of 20×. All the obtained MPS genotypes were consistent with the Sanger results listed in Table S3-2. Figure 2(A) illustrates the coverage depth statistics obtained at the six libraries. The mean coverage depth of S1 (10 ng) was 5493× ± 2578, while of S6 (0.2 ng) was 744× ± 473.6. For sample S6 (0.2 ng), the highest coverage reads was 3 175× while the lowest was 45×. Figure 2(B) shows the performances of mean *F*_MAR_ values of the 221 SNPs at the six different concentrations. The allelic balance of heterozygotes varied more in experiments with lower amounts of DNA as expected. For sample S1 (10 ng) and S2 (5 ng), all SNPs can be accurately genotyped with ideal *F*_MAR_ values; for sample S3 (2 ng), three heterozygotes showed a little bit allelic imbalance (*F*_MAR_ values 60%–62%). However, when DNA ranges from S4 (1 ng) to S6 (0.2 ng), more and more SNPs were detected with *F*_MAR_ values between 60% and 90%, and some of them were hard to define accurate genotypes. Results illustrated that at least 1 ng initial DNA should be prepared for library construction with this custom assay.

### Mixture testing

**Figure 3.**
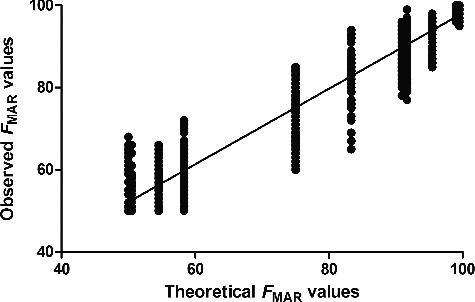
Plotting profiles of theoretical *F*_MAR_ values and observed *F*_MAR_ values with different ratios of mixtures (*R* ^2^ = 0.933 1). Ten problematic SNPs in Table 1 were excluded.

Seven mixtures of two references (9947A and 9948) ranging from 1:1 to 1:100 were made and each mixture was sequenced twice on Ion 316 chip. Table 2 lists the theoretical *F*_MAR_ values of different ratios where the genotypes of the two individuals differed. Figure 3 shows the theoretical *F*_MAR_ and observed *F*_MAR_ values of mixtures with genotypes mentioned in Table 2. The 10 poorly performing SNPs listed in Table 1 were excluded from this analysis also. There was a clear linear correction between the expected and observed *F*_MAR_ values (*R* ^2^ = 0.9331), which indicates that the assay generated a loyal representation of DNA samples. Most of the noise ratio data of the two control samples (listed in Figure S1) in many loci were lower than 1%, making them possible to identify mixtures of 1:100 with a high degree of certainty if the total reads was high enough.

**Table 2.** Theoretical *F*_MAR_ values for different ratios of mixtures (except the two contributors with same genotypes).

Genotype 1	Genotype 2	Mixture ratio	Formula for calculation of theoretical *F*_MAR_	Theoretical value of *F*_MAR_
aa	bb	1:1	2a/(2a + 2b)	50.00
aa	bb	1:5	10b/(2a + 10b)	83.33
aa	bb	5:1	10a/(10a + 2b)	83.33
aa	bb	1:10	20b/(2a + 20b)	90.91
aa	bb	10:1	20a/(20a + 2b)	90.91
aa	bb	1:100	200b/(2a + 200b)	99.01
aa	bb	100:1	200a/(200a + 2b)	99.01
aa	ab	1:1	3a/(1b + 3a)	75.00
aa	ab	1:5	7a/(7a + 5b)	58.33
aa	ab	5:1	11a/(11a + b)	91.67
aa	ab	1:10	12a/(12a + 10b)	54.55
aa	ab	10:1	21a/(21a + b)	95.45
aa	ab	1:100	102a/(102a + 100b)	50.50
aa	ab	100:1	201a/(201a + b)	99.50
ab	bb	1:1	3b/(1a + 3b)	75.00
ab	bb	1:5	11b/(a + 11b)	91.67
ab	bb	5:1	7b/(5a + 7b)	58.33
ab	bb	1:10	21b/(a + 21b)	95.45
ab	bb	10:1	12b/(10a + 12b)	54.55
ab	bb	1:100	201b/(201b + a)	99.50
ab	bb	100:1	102b/(102b + 100a)	50.50

### Forensic application

Fifty unrelated individuals (34 males and 16 females) of Chinese Han population were sequenced twice with the custom panel on Ion 318 chip. Forensic parameters of the 221 SNPs (the 10 poorly performing SNPs were excluded) were analyzed with Arlequin 3.5 [22], PowerMarker v3.25 [23] and SNPAnalyzer. No significant deviation from HWE was detected in the distribution after Bonferroni correction. Allelic frequencies and forensic parameters of the 221 SNPs are listed in Table S4. No SNPs were observed with MAF below 0.1. Four SNPs (rs826472, rs1355366, rs938283 and rs733164) were detected with MAF between 0.1 and 0.2. DP values range from 0.211 2 (rs733164) to 0.5000, while PIC values range from 0.1889 (rs733164) to 0.3750. No significant LD was observed with all unique pairings; thus, the cumulative discrimination power (CDP) was 1–1.49E−62 and cumulative probability of exclusion (CPE) for duo cases was 1–2.98E−12 while for trios was 1–3.51E−23.

## Discussion

The newly developed panel we present here allows simultaneous sequencing of 231 SNPs chosen from SNPforID [11], IISNP [12], HapMap, dbSNP and published literatures [13–20] for forensic application. The library length ranges from 125 to 175 bp, which is suitable for sequencing of degraded DNA. The performance of the custom SNP panel and Ion Torrent PGM was tested by studying SNP sequencing concordance with traditional Sanger sequencing, individual sample sequencing, sensitivity and mixtures testing.

In this study, HID_SNP_Genotyper.42 (v4.2) plug-in was mainly applied for MPS–SNP genotype calling. Sometimes, it interpreted the sequence data wrongly and gave incorrect genotypes [24,25]. Thus, parameter of *F*_MAR_ was adopted for allelic performance of targets. *F*_MAR_ should be 50%–60% for accurate heterozygotes calling and above 90% for homozygotes and Y-SNPs [9], which is comparable to the heterozygote balances of STRs typed with PCR–CE [2,9,24,25]. With concordance study, all available MPS genotype calling were fully consistent with Sanger sequencing results across the two control samples of 9947A and 9948; even several heterozygotes were detected with *F*_MAR_ values above 60%. And with the 50 sequenced individuals, seven SNPs (rs4606077, rs334355, rs430046, rs2920816, rs4530059, rs1478829, and rs1498553) were detected with mean *F*_MAR_ values at heterozygotes of 60%–90%. Inconsistent *F*_MAR_ values were always observed at them. No exact reason was obtained to explain this phenomenon. Primer re-designing of them or exclusion from the assay may improve the whole performance of the custom MPS–SNP panel.

Genotypes at SNP rs1498553 of 9947A and at rs1498553, rs1478829 and rs7141285 of 9948 were labelled with “NN” as observed coverage depth below the analytical threshold of 20×, indicating that the primer efficiency for library construction was too low. With the 50 tested individuals, two more SNPs (rs12714757 and rs2189011) were detected with mean coverage reads below 100× (Table 1). With these SNPs, increasing the primer concentration accordingly in the primer mix may greatly improve the SNP performance. The 10 SNPs were listed as problematic SNPs and excluded from data analysis, because of the sequencing difficulties such as ambiguous base calling or insufficient amplification efficiency. Coverage variation was observed among the remaining 221 SNPs and each SNP generally showed similarly high or low coverage across the tested samples, which is directly in association with the primer efficiency.

With sensitivity testing, accurate genotypes were readily obtained from 10 to 2 ng with recommended library preparation condition. With modified library-PCR conditions, sequencing of 1 ng DNA was able to accurately genotype the targets with a high degree of coverage and an ideal allelic performance. The allelic balance suffered when the amount of input DNA was lower than 1 ng; in other words, the optimal amount of input DNA in the custom MPS–SNP panel was above 1 ng. From Figure 2, it is likely that the sensitivity may be improved by further optimization of library construction or by removing some SNPs from the panel.

We applied parameter of *F*_MAR_ to estimate the SNP performance of mixtures. MPS data can give coverage reads at each bases at each position in detail, providing a more secure basis for analyzing mixtures [24–26]. Considering a 1:1 mixture, the theoretical *F*_MAR_ values would become 50% (aa:bb) or 75% (aa:ab or ab:bb) (Table 2). The performance of the *F*_MAR_ values would indicate that the sample is a mixture. Detection of mixtures is possible by analysis of the *F*_MAR_ values in most loci with high coverage depth and low level of noise. However, since similar allelic imbalance will be observed at involved SNPs, it is essentially impossible to deconvolute a mixture unless the precise mixture ratio is known, which is unlikely in real case samples. Study of Børsting et al. [26] indicated that mixtures with almost equal amount of DNA from the major and minor contributors can be identified using the parameters of Hb, and the mixtures with less contribution from the minor contributor can be identified due to increased noise, which is same with our finding. All results indicated that MPS–SNP data are useful for mixture analysis; however, more tested samples should be involved and more convenient data interpret tool should be explored.

With forensic application, the CDP of the 221 independent auto-SNPs was 1–1.49E−62 and CPE for duo cases was 1–02.98E−12 while for trios was 1–3.51E−23. Above SNPs constitute an excellent panel for individual identification including paternity testing, and the unlinked status of the subset of SNPs we have identified also makes them useful for complex kinship analysis.

## Supplementary Material

1281011_supplementary.zip
